# The interplay between endometriosis and fertility in rats: a systematic review

**DOI:** 10.25122/jml-2021-0329

**Published:** 2022-06

**Authors:** Dimitrios Kanellopoulos, Dimitra Karagianni, Vasilios Pergialiotis, Grigorios Patsouras, Konstantinos Patsouras, Nikolaos Nikiteas, Andreas C Lazaris, Dimitrios Iliopoulos

**Affiliations:** 1 Laboratory of Experimental Surgery and Surgical Research N.S. Christeas, National and Kapodistrian University of Athens, Athens, Greece; 2 Department of Obstetrics and Gynecology, Tzaneio Hospital, Athens, Greece; 3 1^st^ Department of Pathology, National and Kapodistrian University of Athens, Athens, Greece; 4 2^nd^ Propaedeutic Department of Surgery, National and Kapodistrian University of Athens, Athens, Greece

**Keywords:** endometriosis, rats, subfertility, infertility

## Abstract

For the last decades, endometriosis has been a major gynecological problem and a significant cause of infertility for women worldwide. It is estimated that the disease affects about 10–15% of all women of reproductive age and 70% of women suffering from chronic pelvic pain. At the same time, the incidence is about 40–60% in women with dysmenorrhea and 20–30% in women with subfertility. Despite the high percentage of affected women, endometriosis is still characterized by insufficient knowledge of the pathogenic processes, leading to the development and continuity of the disease. For this reason, there is a significant need for insight and understanding of the pathogenesis of endometriosis. This systematic review aims to present the latest data on the use of rats in endometriosis research and to explore how fertility is affected in rats with endometriosis. The methodology included a review of the available publications retrieved by a search in various scientific databases, such as PubMed, Scopus, Medline, and Google Scholar. The initial search generated 30 titles, with 10 articles fulfilling the inclusion criteria. In conclusion, several surgical techniques have been proposed to induce endometriosis, mainly using rats as the appropriate animal model. Studies in rats showed that endometriosis causes infertility and that pregnancy rates are lower for rats with endometriosis than those without endometriosis. In addition, rats with endometriosis have significant abnormalities in the structure of their oocytes as well as in the development of their embryos (genetic abnormalities).

## INTRODUCTION

Endometriosis has been a major cause of infertility and the most common cause of chronic pelvic pain for a significant number of women worldwide [1–8]. Epidemiologically, it is estimated that the disease affects about 10–15% of all women of reproductive age and 70% of women suffering from chronic pelvic pain. At the same time, the incidence is 40–60% in women with dysmenorrhea and 20–30% in women with subfertility [1, 3, 6, 7]. The cause of the disease is multi-parametrical, and exposure to menstruation (early menarche and late menopause) appears to increase the risk of endometriosis [3–5]. Furthermore, genetic and environmental causes seem to play an essential role in the pathogenesis of the disease [7–9].

The disease has a typical onset during early puberty, with an early age of menarche and acute pelvic pain that lasts during menstruation (sometimes, even days before or after, with cyclic and acyclic pain). Over the years, other symptoms result, according to the number of organs involved and the extent of the endometrial tissue covering them [1, 4]. Three main theories have been proposed thus far to explain the pathophysiology of the disease: (a) retrograde menstruation; (b) coelomic metaplasia; and (c) müllerian remnants [7–11]. The most dominant of the three is the retrograde menstruation hypothesis [1, 7–11]. However, none have provided a clear and adequate explanation of how the different types of endometriosis could occur. According to this theory, fragments of the normal uterus endometrium gradually move to reach the pelvis via transtubal retrograde flow, following its implantation into the peritoneal cavity and the abdominal organs. Furthermore, the implanted tissues (whose functions are typically estrogen-dependent) start proliferating in these ectopic places, which causes acute pelvic pain and symptomatology connected to the organs most affected (dysmenorrhea, dyspareunia, dysuria, pain during ovulation, bowel and/or urinary bladder -related pain) [1, 7, 12–21].

Despite the high percentage of affected women, it is still characterized by insufficient knowledge of the pathogenic processes, leading to the development and continuity of the disease. For this reason, there is a significant need for knowledge and understanding of the pathogenesis of endometriosis. For ethical reasons, controlled experiments are not allowed in women with endometriosis. However, experiments are essential in animal models [1–7, 12–21].

The purpose of the current systematic review was to research how fertility is affected in rats with endometriosis.

## MATERIAL AND METHODS

**Figure 1:**
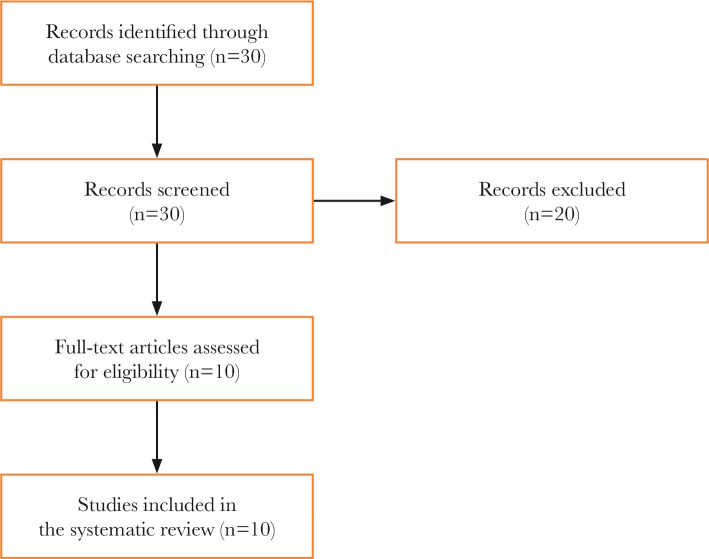
Flow diagram of the search and selection process.

The methodology included a review of the available publications retrieved by a search in various scientific databases, including PubMed, Scopus, Medline, and Google Scholar. The most recent worldwide bibliographical references were studied, and the results were recorded chronologically (from the oldest to the most current ones). The research was based on inclusion criteria: (a) studies including rat models used to study endometriosis and subfertility; (b) full text available in English; and (c) period of publication between 1985 and 2020. The following search terms were used: "endometriosis", "rats", "subfertility", and "infertility", with the initial search generating 30 titles. Titles and abstracts were examined for relevance to the objective of the review. Following the assessment of the titles and abstracts, 20 publications were excluded as irrelevant to the study's objective. In the end, 10 articles remained for inclusion (Figure 1).

## RESULTS

The initial search generated 30 titles. Titles and abstracts were examined for relevance to the review objective. After assessing the titles and abstracts, 20 references were excluded because they were apparently not relevant to the study's objective. Finally, 10 articles were included in the review (Figure 1).

Stating the bibliographical references in chronological order, in November 1985, Vernon & Wilson published a study about possible ways of surgical induction of endometriosis in rats [22]. The article described three models of inducing endometriosis in rats. First, four uterine squares were sutured to the peritoneal cavity; second, uterine luminal lavages were instilled into the peritoneal cavity; third, endometrial scrapings were flushed into the peritoneal cavity, followed by a control group (sham-operated controls). In the days after the surgery, rats were examined for the presence of endometrial implants both in the pregnant and non-pregnant groups. The results showed that the peritoneal adhesions were greater in rats with induced endometriosis than in the normal (sham-operated) controls, which could partially explain the infertility in rats with endometriosis. Adhesions that occur with endometriosis can block the fallopian tubes or uterus, making it difficult for the sperm to meet the ovum. However, no difference was seen regarding the severity of adhesions between pregnant and non-pregnant rats. Rats with endometriosis showed a statistically significant decrease (p<0.05) of pups delivered at term by 48% [22].

Five years later (May 1990), Rajkumar et al. published an article describing the effects of pregnancy and ovariectomy on the growth of endometrial implants in rats with experimentally induced endometriosis [23]. Results showed that rats who had undergone endometriosis in the lab were as fertile as the control group and that endometrial cells can survive where they have been implanted and grow into a new implant in the future, even in cases where no lesions could be seen [23].

In December 1992, Barragán et al. published another study examining the effects of pregnancy and lactation on the ectopic endometrial tissue, as well as the fertility capacities, in rats that had experimental procedures to achieve endometriosis [24]. The study included 44 rats with endometriosis induced in the lab and 41 normal controls, and the results showed reduced pregnancy rates for rats with endometriosis (65.7%), in contrast to the control group (100%). Moreover, during the lactation period, the implants in both groups showed no difference in their growth, thus implying that the absence of estrus during lactation proves beneficial to the rats suffering from endometriosis [24].

Next, in May 1995, Cummings & Metcalf published another study examining the role of environmental chemicals in the progression of endometriosis in rats [25]. Experiments were conducted (slices of the uterus were sutured to intestinal mesenteric vessels) to investigate (a) how 2, 3, 7, 8-tetrachlorodibenzo-p-dioxin (TCDD) could promote endometriosis in rats, (b) how endometriosis and immunodeficiency are linked together, and (c) if and to what extent humoral immunity is suppressed in mice – but not rats – after being exposed to TCDD. The results showed that the growth of the lesions in the implantation sites was clearly hormone-dependent, thus offering a new model on the ability of specific xenobiotics to promote endometriosis in rats. This model seems appropriate for studying xenobiotic-induced damage to primordial follicles that can result in early ovarian failure (premature menopause) [25, 26].

In March 2002, Sharpe-Timms published another paper on how rats could be used as a research model to study endometriosis. The paper states that rats can be very useful for the deeper in vivo study of the pathogenesis and pathophysiology of endometriosis and in the study of novel therapeutic approaches that are not suitable to be tested in humans [27]. Furthermore, the research proposes that rats can be a good animal model because rat endometriotic tissues are similar to human endometriotic tissues. The first surgical method mentioned is the autotransplantation of uterine squares into the peritoneal cavity, followed by the monitoring of the progression of the transplantation and the clinical symptomatology in vivo. Rats with endometriosis display similar symptoms to humans, with rats showing reduced fertility and fecundity. Another important cue to the similarity of rat and human endometriosis pathophysiology is gene expression and protein production. For these reasons, the study encourages the use of rats as animal models and promises optimistic results for the near future [27].

In November 2006, Mohammadzadeh et al. published another article on how endometriosis could be induced by implanting the endometrial fragments in rats. This article describes 2 groups of rats, (1) rats auto transplanted with endometrial tissues and (2) rats auto transplanted with fat tissue (control group), monitored after surgery (autotransplantation) for the clinical impact of the transplants and their development [28]. The results showed that after surgically induction of endometriosis in rats, only endometrial (and not fatty) implants grew as a cystic structure inside the rats' bodies, probably because of the endometrial cell's unique capacity to promote the formation of such lesions in the peritoneal cavity. Adhesions were detected in 7 out of 10 rats suffering from endometriosis and 2/10 of the rats in the sham group. The number of the estrous cycles was similar in both groups [28].

In April 2009, Stilley et al. published a study that examined the role of tissue inhibitors of metalloproteinase 1 in the reduced fecundity of rats with endometriosis. Even though the study does not present specific surgical techniques, it offers a significant explanation of how the expression of matrix metalloproteinases and their tissue inhibitors by ectopic and eutopic endometrium could play an important role in the pathogenesis of endometriosis [29]. The research compared rats with endometriosis with normal controls. The results show that rats with endometriosis had significant abnormalities in the structure of their oocytes and the development of their embryos (genetic abnormalities) before the implantation. The study suggests that one reason for these abnormalities in the endometriotic rats might be that they have more tissue inhibitor matrix metalloproteinase 1 (TIMP1) in their peritoneal fluid compared to normal controls. It also proposes that endometriotic lesions might lead to permanent epigenetic changes in the rats born from these endometriotic mothers [29].

In January 2012, Pelch et al. published a similar article about the use of rats as animal models for endometriosis, suggesting that some of the most important advantages of rats are the cost-effectiveness and availability (wide variety of transgenic mice) [30]. The study proposes a surgical technique of transplanting endometrium from the uterus to the intestinal mesentery (experiment first carried out on rats and, later, on mice) of the same animal, which is an effective way of surgical insertion of endometriosis in mice. This rodent model of surgically induced endometriosis demonstrates many similarities to the disease in humans, including reduced fertility and fecundity and altered gene and protein expression [30].

In January 2015, Edson Ximenes Gomes Pereira et al. published a similar study about an animal model that could be used to understand the pathophysiology of endometriosis and the effects of certain pharmacological agents on endometriotic animals [31]. The study used 53-month-old rats that were divided into four groups according to the pharmacological agent with which each was treated: (1) the estradiol group, (2) the medroxyprogesterone acetate group, (3) the triptorelin pamoate group, and (4) the acetylsalicylic acid [17]. All the groups were subjected to surgically induced endometriosis, and the progression of the transplants was monitored on days 1, 7, 14, and 21. The results showed that the weight of the endometrioma and hemiuterus was linked to the pharmacological agents provided to the animals. Moreover, the experimental model of subcutaneous endometriosis is a good option for researchers due to its reproducibility, cost-effectiveness, and facile implementation [31].

Lastly, in February 2020, Dera-Szymanowska et al. published another interesting article suggesting that immunomodulation (through alteration of the potentially pathogenic gene expression profiles of Bax, Tert, and Mki67 genes in the endometrial cells of the rats) seems to be able to inhibit the development of endometriosis at a substantially important level, thus moving one step forward from the implantation of the lesions, onto how these lesions could be reduced (in size and functionality) in vivo [32].

## DISCUSSION

The probability of conception in women with mild endometriosis ranges between 2–5%, in contrast to 15–25% in healthy fertile women [33–36]. The correlation between endometriosis and infertility is therefore evident. The pathogenesis of infertility in women suffering from endometriosis is multifactorial and difficult to explain. Many animal models have been used to offer insight into the mechanisms through which endometriosis causes infertility [22–32, 37, 38].

The first experiments on endometriosis were conducted on primates in the 1950s. In an attempt to simulate retrograde menstruation, the cervix of the female monkey was surgically repositioned in such a way as to cause endometrial lesions [37, 38]. The experimental cost of these animals was excessive. Therefore, small laboratory animals are currently used to study endometriosis (*i.e*., rats) [37, 38]. Human and rat reproductive physiology have relevant differences. Hence, these models possess limitations. Because rodents do not menstruate, they do not develop spontaneous endometriosis, and therefore the disease has to be induced artificially by the autotransplantation of the uterine tissue [37]. Unlike human beings and non-human primates, other animal models do not develop endometriosis spontaneously. However, researchers can induce endometriosis in these organisms through the ectopic transplantation of endometrial tissue [22–32, 37, 38]. Despite these limitations, rat models offer significant advantages, such as the limited costs and the opportunity to perform studies in large groups of genetically similar animals [37]. Furthermore, rat models of surgically induced endometriosis demonstrate many similarities to the disease in humans, including reduced fertility, fecundity, and altered gene and protein expression [27]. For these reasons, our research encourages using rats as animal models to study the correlation between endometriosis and infertility.

## CONCLUSION

In conclusion, according to all the studies above, it seems that endometriosis is a serious gynecological disease that can significantly reduce the patients' life due to both the acute, chronic pelvic pain and the subfertility issues it brings upon women of reproductive age. For these reasons, several surgical techniques have been proposed to induce endometriosis, mainly using rats as the appropriate animal model. There are many reasons why rats and rodents are used in these experiments. Firstly, due to the high reproducibility of the research, and secondly, due to low financial expenditure. The surgical techniques proposed include autotransplantation (transplants from the same rat to the same rat), and the results seem to be promising for the future, for the representation of the pathophysiology of the disorder, primarily in an animal model, and subsequently (with respective biological processes) in humans. Studies in rats showed that endometriosis causes infertility. Pregnancy rates are lower for rats with endometriosis (65.7%) than those without endometriosis (100%). Rats with endometriosis had significant abnormalities in the structure of their oocytes and the development of their embryos (genetic abnormalities). Gene therapies and pharmacological agents play an important role when the appropriate therapeutic steps are taken, which results in a significant percentage of endometriomas being able to shrink substantially. In these ways, endometriosis can hopefully be well defined, studied, and clearly understood as a network of biological mechanisms in rats and humans, thus providing the scientific world with important information and contributing to the reduction in the rates of this disease in future years.

## Data Availability

Further data is available from the corresponding author on reasonable request.
